# ContraDRG: Automatic Partial Charge Prediction by Machine Learning

**DOI:** 10.3389/fgene.2019.00990

**Published:** 2019-10-30

**Authors:** Roman Martin, Dominik Heider

**Affiliations:** ^1^Department of Mathematics and Computer Science, University of Marbug, Marburg, Germany; ^2^Department of Organic-Analytical Chemistry, TUM Campus Straubing, Straubing, Germany

**Keywords:** PRODRG, ATB, machine learning, molecular dynamics simulations, partial charge prediction

## Abstract

In recent years, machine learning techniques have been widely used in biomedical research to predict unseen data based on models trained on experimentally derived data. In the current study, we used machine learning algorithms to emulate computationally complex predictions in a reverse engineering–like manner and developed ContraDRG, a software that can be used to predict partial charges for small molecules based on PRODRG and Automated Topology Builder (ATB) predictions. Both tools generate molecular topology files, including the partial atomic charge, by using different procedures. We show that ContraDRG can accurately predict partial charges in a fraction of the time, because it exploits existing complex models with intensive calculations by using machine learning techniques and thus can also be applied for screening projects with large amounts of molecules. We provide ContraDRG as a web server, which can be used to automatically assign partial charges to incoming user-specified molecules by using our machine learning models. In this study, we compared ContraDRG with PRODRG and ATB in regard of predictivity by statistical methods. ContraDRG allows predicting ATB-derived partial charges with an *R^2^* value up to 0.980 and for PRODRG up to 1.00. While ATB requires hours or days for the quantum mechanical accurate calculation and refinements, ContraDRG does its approximation within seconds.

## Introduction

In the last decades, several studies demonstrated how machine learning algorithms were able to create accurate predictions or classifications from experimentally derived data. The applications of machine learning algorithms in biomedical research are diverse (Larrañaga et al., 2006) and range from single-molecule interaction prediction for drug design (Lavecchia, 2015) or omics pattern recognition (Stanke and Morgenstern, 2005), toward the prediction of entire biological systems (D’Alche-Buc and Wehenkel, 2008).

However, in the current study, we used machine learning algorithms to emulate computationally intensive calculations. Precise determination of topology parameters for small molecules, particularly partial charges, is a crucial step for molecular dynamics (MD) simulations and other biochemical and biophysical computations. In particular, MD simulations depend heavily on the accurate parameterization of the molecules; otherwise, the simulations tend to be unreliable and misleading (Lemkul et al., 2010). One main challenge for generating reliable predictions is the ability to create a force field consistent topology for new small molecules since the force fields theory is mostly derived from empirical analysis.

For this purpose, there are different force fields available, based on diverse parameters and underlying theories, such as GROMOS (van Gunsteren et al., 1996; Daura et al., 1998; Scott et al., 1999; Schuler et al., 2001; Oostenbrink et al., 2004), OPLS (Jorgensen and Tirado-Rives, 1988; Jorgensen et al., 1996), CHARMM (Patel and Brooks, 2004; Patel et al., 2004), and AMBER (Cornell et al., 1995; Wang et al., 2004). Parameterization for synthetic small molecules is supported by the general AMBER force field (Wang et al., 2004) and the general CHARMM force field (Patel and Brooks, 2004; Patel et al., 2004), in contrast to GROMOS and OPLS. While detailed information about the GROMOS96 parameter sets is not publicly available, OPLS-AA reveals their entire parameter sets, which includes geometry optimization and quantum chemical calculations (Jorgensen et al., 1996; Kaminski et al., 2001). Thus, users of the GROMOS96 force field rely on empirical parameters and subsequent validations by thermodynamic integration (Oostenbrink et al., 2004).

Over the last years, some freely available tools were developed, refined, and established for automated topology generation. Two commonly used tools are PRODRG (Van Aalten, 1996; Schüttelkopf and Van Aalten, 2004) and the Automated Topology Builder (ATB) (Malde et al., 2011; Koziara et al., 2014; Stroet et al., 2018). Both are frequently used tools that receive user-defined small-molecule files and return parameterized GROMOS-compatible topology files including their partial atomic charges. While PRODRG partial charge determination is based on mapping of building blocks and charge groups onto a database, ATB uses quantum chemical calculations involving electron densities and geometry optimizations (Chandra Singh and Kollman, 1984). However, PRODRG is much faster compared to ATB and produces topologies within seconds, while ATB requires up to multiple days, but generates more precise, more reliable, and more consistent results (Lemkul et al., 2010; Malde et al., 2011). Both tools have been already used for protein–peptide, protein–ligand, protein–lipid, and pharmaceutical drug optimizations (Santos et al., 2017). Although both tools provide free access for automated file parameterization, only ATB supplies a modern application programming interface. Additionally, there are several stand-alone tools, such as Open Babel (O’Boyle et al., 2011) and AutoDock Tools (Morris et al., 2009), which can predict partial charges based on different methods like MMFF94 (Halgren, 1999), based on quantum chemical calculations, or QTPIE (Chen and Martı, 2007), which describes the flow in molecules based on charge transfer variables.

While PRODRG and ATB are proprietary software, they do provide free access for academic purpose. Contrary to that, fully proprietary software like VeraChem’s VCharge or Schroedinger’s Maestro, which predict, among others, partial charges are also available. VCharge uses a method based on QM-derived electronegativity equalization (Gilson et al., 2003), and Maestro computes the charges according CM1A-BCC (OPLS3e) (Marenich et al., 2012; Roos et al., 2019). Additionally, there is proprietary software such as Amber that requires external tools for partial charges predictions, like the provided and recommended free antechamber (Wang et al., 2006). Antechamber applies usually the AM1-BCC method (Jakalian et al., 2002) for small molecules and can be optimized with provided QM calculations by the RESP method (Bayly et al., 1993).

Engler et al. (2019) showed recently in an innovative approach how to solve two common problems of partial charge determination: (i) the single partial-charge assignment per atom and (ii) the total charge determination. By transferring these problems into a multiple-choice knapsack problem (Dudziński and Walukiewicz, 1987; Kellerer et al., 2004), they were able to predict the partial charges automatically. Moreover, a recent study showed that machine learning prediction based on quantum-chemical calculation can be used to predict partial charges (Bleiziffer et al., 2018).

In the current study, we used small-molecule three-dimensional structures files for prediction of partial charges, based on machine-derived data from the web tools PRODRG and ATB. To this end, we analyzed and compared a set of different machine learning methods and emulated the aforementioned tools. Finally, we compared our predictions with the existing tools. This study demonstrates the usefulness of machine learning models for reverse engineering of costly calculations, which are provided in an easy-to-use online tool.

## Materials and Methods

### Dataset

This study is based on two different datasets, namely, the PRODRG dataset and the ATB dataset. The PRODRG dataset is based on randomly selected molecule structures from the PubChem database (Kim et al., 2018). These molecules were converted into Protein Database Bank format *via* Open Babel (O’Boyle et al., 2011) and subsequently predicted *via* the PRODRG server (v. AA100323.0717). Energy minimization was deactivated, and full charge prediction and chirality enabled. The ATB dataset was collected from the curated molecule and topology files from the ATB (v. 3.0) database (Stroet et al., 2018). We mapped the partial charge predictions from the topology files with the provided all-atom Protein Database Bank files.

We calculated the pairwise Tanimoto similarity coefficient *via* Open Babel (linear seven atoms fragments) for all files to ensure that a diverse set of molecules was used (Kim et al., 2018). The Tanimoto coefficient represents a known indicator for molecular structure similarities (Bajusz et al., 2015). Therefore, we determined the coefficient by comparing every molecule to each other. The resulting coefficients were drawn into a violin plot.

### Feature Encoding

**Figure 1:**
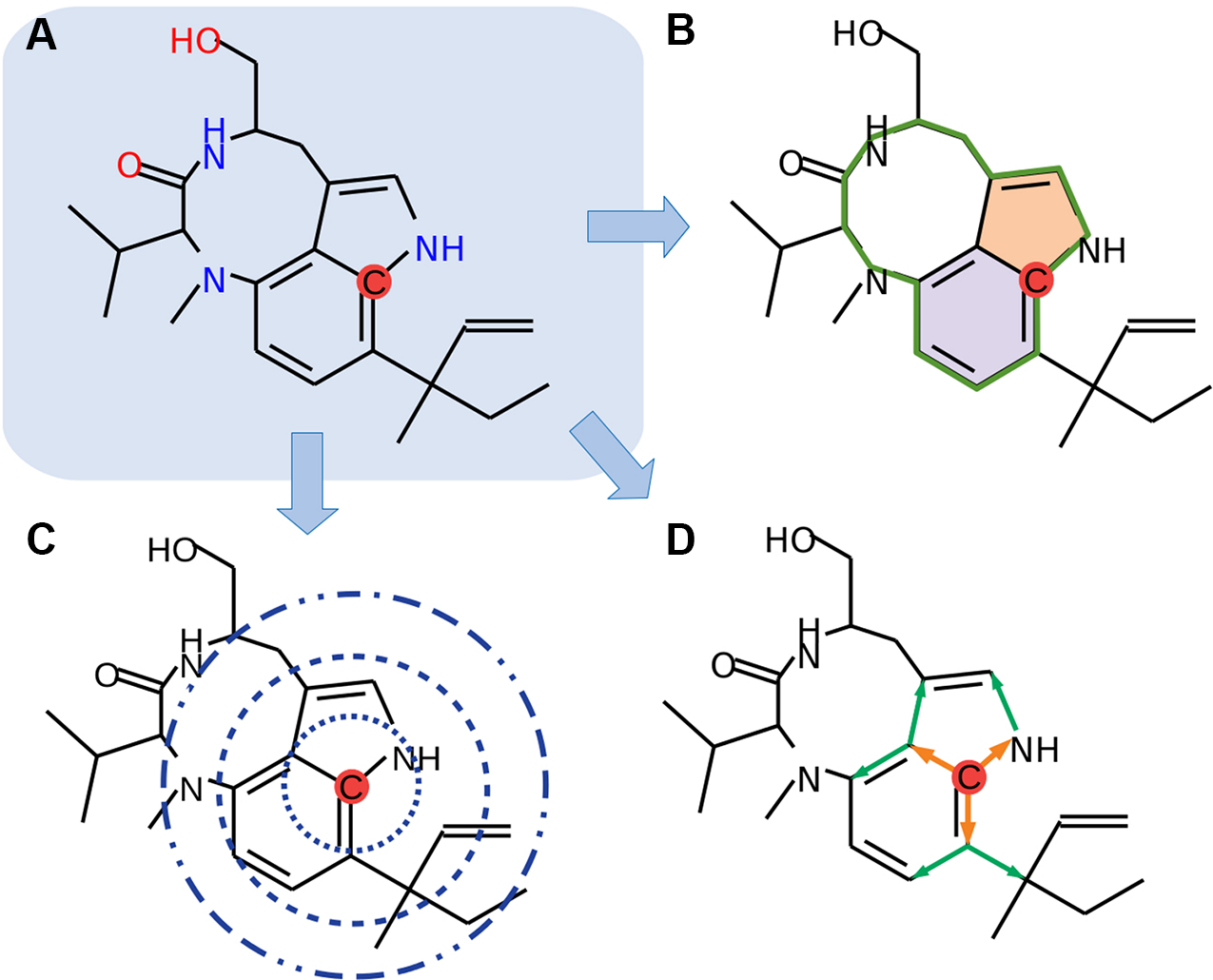
Schematic overview of the feature encoding. **(A)** Each atom will be selected (red dot), and encodings will be generated **(B**–**D)**. **(B)** Overall circular structures (green line) and nested (colored areas) are detected by a depth-first search. **(C)** Distance searches with three different radii are applied. **(D)** Second-level neighbors path tracing is implemented (orange arrows, first level; green arrows, second level). Chemical structures were drawn with MolView (https://molview.org).

In the current study, we focused only on organic elements, namely, carbon, hydrogen, nitrogen, oxygen, phosphorus, sulfur, fluorine, bromine, and iodine (C, H, N, O, P, S, F, Cl, Br, and I). We used 61 different features for the encoding of the molecules, where all atoms are individually analyzed (**Figure 1**). Molecules are internally represented as a cyclic undirected graph, where atoms correspond to vertices, and bonds to edges. These encodings include the hybridization state of carbon atoms, sizes and amounts of nested circles, distances to adjacent atoms, and presence of neighbors through a second-level path tracing. Nested circular structures were identified by a depth-first search derived from the graph theory.

To encode an entire molecule, a list of the positions of the atoms and an adjacency matrix for the bonds are necessary. Protein Database Bank files and SMILE (Weininger, 1988) files can be encoded in such a way easily. However, in contrast to existing approaches, we take explicitly the three-dimensional information into account, thus allowing making prediction also for theoretical molecules.

### Machine Learning

We used the R package caret (v. 6.0-81) (Max and Kuhn, 2008) for building the machine learning models. We build models for each element independently. The datasets (one dataset for each element) were split into train and test data with a ratio of 1:4. We trained different models including linear regression, stochastic gradient boosting (Friedman, 2002), random forests (RF) (Breiman, 2001), quantile regression forests (Meinshausen, 2006), weighted k-nearest neighbors (Altman, 1992), and support vector machines (SVMs) (Cortes and Vapnik, 1995) with different kernels. RFs were trained with 500 trees and k-nearest neighbors were built based on *k=* 7 and a Minkowski distance of 2. All other models were trained with default parameters. All models were trained with the partial charge values as labels from PRODRG or ATB, respectively. The models are evaluated based on root median square error (RMSE):

(1) $$\mathrm{RMSE}=\sqrt{\frac{\Sigma_{T}^{t=1}{\left({\hat{y}}_{t}-y_{t}\right)}^{2}}{T}}$$

Furthermore we used the normalized RMSE:

(2) $$\mathrm{NRMSE}=\frac{\sqrt{\frac{\Sigma_{T}^{t=1}{\left({\hat{y}}_{t}-y_{t}\right)}^{2}}{T}}}{\sqrt{{\left(\min(y)-\max(y)\right)}^{2}}}\cdot 100$$

A direct comparison between the different software tools, respectively, the algorithms, is not possible since the applications are using different force fields. However, the aforementioned metrics enable a direct comparison of the machine learning predictions to the original software.

### Molecular Dynamics

We tested the ATB-derived random forest models, with 50 randomly chosen molecules from the ATB database with experimental hydration free energy (ΔG^hyd^). Topologies and coordinate files were obtained by the ATB database. Parameters for the molecule dynamics were taken from the FreeSolv database (Mobley et al., 2009; Mobley, 2013; Mobley and Guthrie, 2014; Duarte Ramos Matos et al., 2017). We used the *gromos54a7_atb.ff* force field according to ATB. Simulations were run under GROMACS (v. 2016.3) with NPT conditions at 298 K and 1 atm. The cutoff for the van der Waals (rvdw) and electrostatic interactions (rcoulomb) was set to 1.2 nm. The simulations were performed with 20 λ-steps and 2 fs per time step, resulting in 12.5-ns simulations per λ-point. GROMACS simulations require removing all nonpolar hydrogens for a united-atoms model. For ContraDRG, original partial charges from ATB were overwritten with ContraDRG predictions. Therefore, we summarized all removed charges into the adjacent remaining atom. Atom-centered partial charge predictions occasionally generate molecules with an excess of net total charges. The excess was eliminated by distributing the excess equally through a molecule. A comparison of the absolute errors between the experimental ΔG^hyd^ free energy and ATB and that between the experimental ΔG^hyd^ free energy with ContraDRG were performed by a Welch *t* test (Welch, 1947). We omitted MD simulations with PRODRG topologies since it has been reported as inaccurate (Lemkul et al., 2010), which could be confirmed in our analyses.

### Web Application

The web application ContraDRG is based on an Apache web server (v. 2.4.29) with PHP (v. 7.2.17) and R (v. 3.4.4) as background services. Incoming data will be filtered and converted by Open Babel (v. 2.4.1) into temporary internal PDB files. ContraDRG reads the PDB structures, performs the feature encoding, and applies the trained machine learning models. The final output will be generated by the Open Babel and remapped with partial charge values predicted by ContraDRG determining partial charge values. A two-dimensional graph of the molecule will be displayed after the machine learning prediction. Missing three-dimensional molecules structures, as provided by SMILES formatted molecules, will be computed by Open Babel as well. The partial charge prediction will be performed by the random forest models for each element, which have been shown to outperform the other models.

## Results

### Overall Approach

The current study aimed to build a reliable and fast prediction model for partial charges. To this end, we used machine learning algorithms to emulate computationally complex predictions in a reverse engineering–like manner and developed ContraDRG, a software that can be used to predict partial charge assignments based on PRODRG and ATB predictions. We collected thousands of randomly selected molecules from PubChem and the ATB database. Finally, we provide the freely accessible web tool ContraDRG, which can be used for partial charge predictions. The resulting predictions provide a reliable approximation of the original tools. However, predictions are carried out in seconds without any user restrictions.

### Datasets

**Figure 2:**
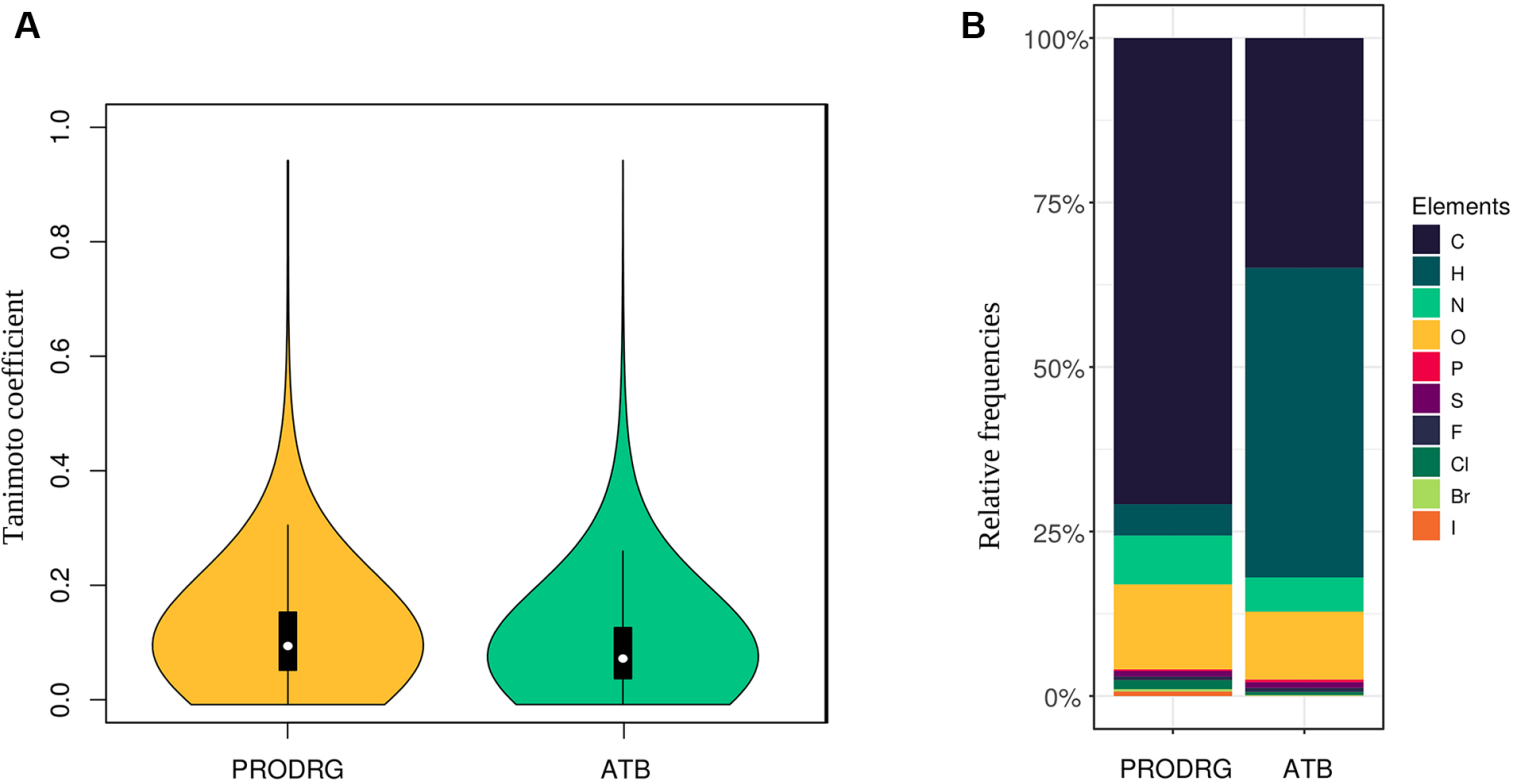
**(A)** The violin plots show the Tanimoto coefficient for both datasets. The plot width correlates with the relative frequencies of the coefficient. The white dot represents the median, while the black box represents the interquartile range, and the black lines, the 95% confidence intervals. One-sample *t* tests for both sets of Tanimoto coefficients show a *p* < 0.001 for a mean below 0.15. **(B)** The distribution of atom types for each dataset is represented by relative bar plots.

We collected 7,000 molecule structures from PubChem with an average size of 19 heavy atoms per molecule (resulting in 132,859 atoms), which were predicted using PRODRG. Seventy percent of the atoms in the PRODRG dataset are carbon, and 13% are oxygen atoms. Moreover, we randomly collected 10,000 molecules from the ATB database with an average size of 25 heavy atoms per molecule. In this ATB dataset, 47% of the atoms are hydrogens, while 35% are carbons. **Figure 2** represents the distribution of all elements in our datasets. Variances in the number of hydrogen atoms between both datasets are due to differences in the underlying model, namely, united-atoms model for PRODRG and all-atoms model for ATB.

To achieve a high variety of different molecules, we analyzed the similarities between every molecule structure to each other by calculating the Tanimoto coefficient in a pairwise manner. The Tanimoto coefficients and their distribution for the PRODRG and the ATB datasets are shown as violin plots in **Figure 2**. The coefficients of all possible pairs of molecules are relatively low, with a median of around 0.11 for the PRODRG and 0.08 for the ATB dataset, indicating a high variance between the incorporated molecules. We used a one-sample *t* test on the Tanimoto coefficients for testing significance against a mean value of 0.15 (*p* < 0.001).

**Figure 3:**
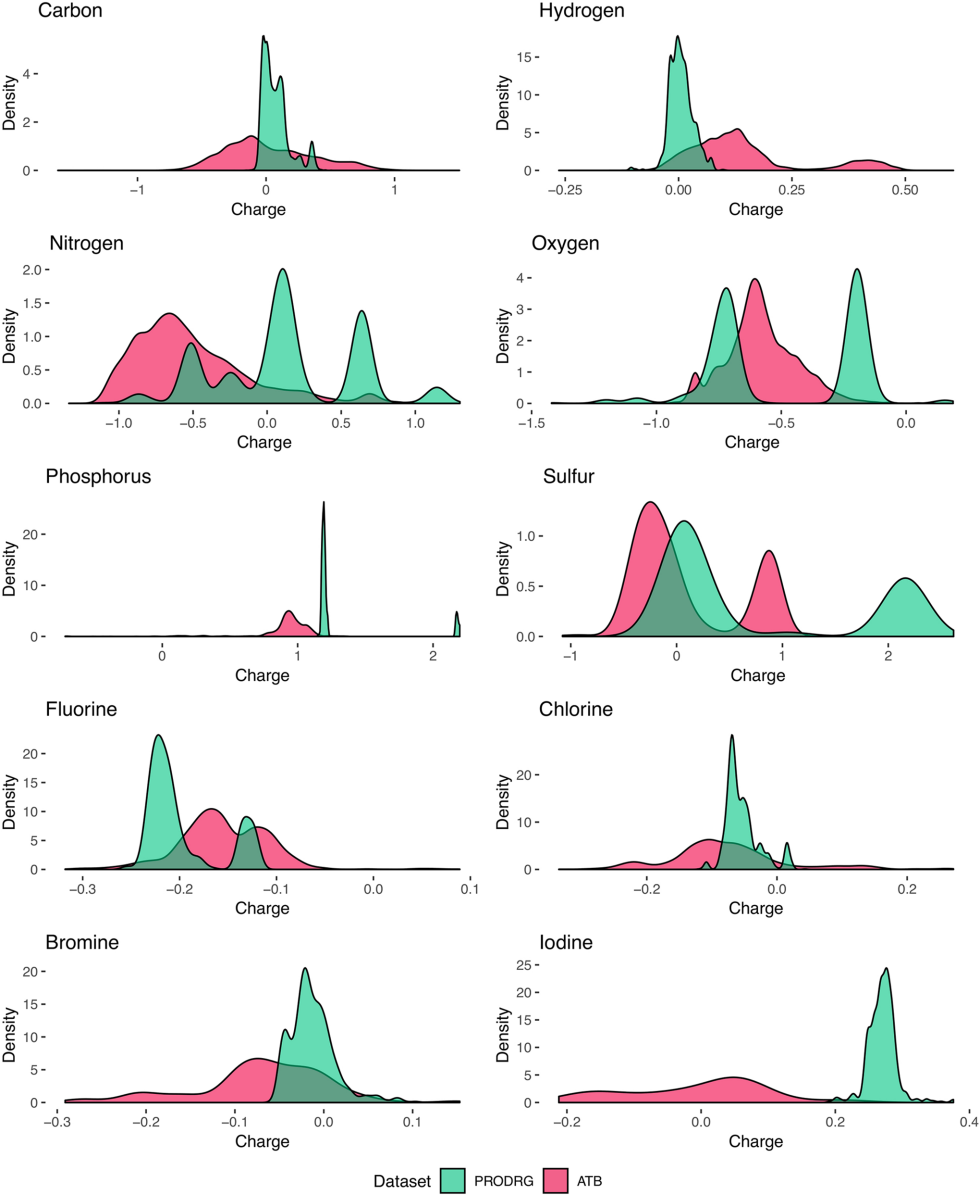
Smoothed kernel density estimates represent the distribution of partial charges (units of *e*) for each molecule in the datasets. Distribution from PRODRGs dataset reveals more clustered peaks (green) than from ATB (red).

Analysis of the charge distribution through all elements shows a variance in the charge predictions between the different datasets in **Figure 3**. Since the occurrence of molecular constitutions and conformations is limited, the partial charges are not equally distributed over the whole range. Moreover, some atoms tend to act as an electron-pair donor, such as oxygen. Therefore, most oxygen is charged negatively or neutral. Generally, the charge predictions differentiate heavily between the PRODRG and ATB datasets. PRODRGs predictions are more clustered than ATB. This clustering can be observed in the shape of the charge distribution curves by the present peaks of the PRODRG dataset in **Figure 3**. One explanation for the highly clustered charges of PRODRG is the fact that PRODRG maps the molecule to a limited set of building blocks and charge groups, while ATB refines partial charges after an initial determination according to the Merz–Singh–Kollman method (Chandra Singh and Kollman, 1984).

### Partial Charge Prediction

We employed several machine learning algorithms for every element on each dataset. Depending on the number of data points, the machine learning algorithm training took several hours up to 10 days on a high-performance cluster, especially for the SVMs and random forest models. Linear regression models turned out to be most inaccurate compared to the random forest models, which mostly outperform all other models in both datasets. For this reason, the ContraDRG web application uses random forest models for the prediction. An exemplary direct side-by-side comparison of ATB-derived ContraDRG prediction with ATB 3.0 is provided in the **Supplementary Material**. For a set of 50 randomly chosen molecules, ATB required an average execution time of 8 h for generating the topology including the partial charges, while ContraDRG required only 9.2 seconds on average for the partial charge prediction per molecule.

**Table 1** represents a shortened overview of the best prediction performance. The full-length table is provided in the **Supplementary Material**. The normalized RMSE values allow an easy comparison for each element since they are normalized to the whole range of present partial charge values. Moreover, the predictions for PRODRG-derived data are more accurate than for ATB, which can be observed particularly for underrepresented elements such as iodine in the ATB dataset. The mean *R^2^* for PRODRG predictions is 0.962 (min. 0.791, max. 1.000) for random forest and 0.685 (0.010–0.985) for SVMs with linear kernel in comparison to the ATB predictions with a mean of *R^2^* 0.908 (0.778–0.982) for random forest and 0.744 (0.520-0.971) for linear SVMs. Overall, the predictions based on the random forest models are more accurate than those based on the other models.

**Table 1:** Performance comparison for partial charge prediction (units of *e*) by random forest and support vector machines with linear kernel of the PRODRG and ATB dataset.

	PRODRG						ATB					
	Random forest			SVM linear			Random forest			SVM linear		
	RMSE	NRMSE	*R*^2^	RMSE	NRMSE	*R*^2^	RMSE	NRMSE	*R*^2^	RMSE	NRMSE	*R*^2^
C	0.011	1.443	0.989	0.054	7.073	0.738	0.069	2.398	0.961	0.152	5.268	0.810
H	0.005	2.878	0.955	0.026	13.924	0.010	0.018	2.313	0.980	0.046	5.794	0.879
N	0.048	1.986	0.990	0.249	10.374	0.730	0.113	5.391	0.919	0.163	7.772	0.834
O	0.051	3.184	0.971	0.153	9.494	0.739	0.047	4.200	0.887	0.071	6.302	0.746
P	0.002	0.152	1.000	0.073	7.157	0.965	0.075	3.712	0.892	0.097	4.803	0.823
S	0.015	0.678	1.000	0.120	5.454	0.985	0.068	3.095	0.982	0.087	3.962	0.971
F	0.003	2.436	0.993	0.007	5.184	0.968	0.017	4.179	0.897	0.037	9.205	0.520
Cl	0.004	2.724	0.980	0.020	15.293	0.415	0.030	5.490	0.895	0.054	9.796	0.705
Br	0.011	8.625	0.791	0.016	12.222	0.589	0.033	8.796	0.778	0.049	13.033	0.531
I	0.004	2.575	0.955	0.010	6.592	0.706	0.036	12.840	0.888	0.062	22.082	0.624
*x¯*)	0.015	2.668	0.962	0.073	9.277	0.685	0.051	5.241	0.908	0.082	8.802	0.744

The root median square error (RMSE) represents the quality of errors while NRMSE shows a normalized RMSE.

The MD analyses show that the predictions of ContraDRG’s ATB-derived random forest models perform as well as ATB in terms of the ΔG^hyd^ free energy calculation. Furthermore, we compared the errors between experimental ΔG^hyd^ values and those derived from ATB with the errors between the experimental data and ATB-derived ContraDRG prediction. No significant differences have been observed by using the Welch *t* test (*p* = 0.53) (Max and Kuhn, 2008). Additional information is provided as **Supplementary Materials**.

## Discussion

In summary, we were able to produce partial charge predictions by our fast and unrestricted approach. Depending on the dataset and the frequency of an element in the dataset, reliable predictions are possible. The models for underrepresented elements such as chlorine, bromine, and iodine performed worse compared to those trained on the most abundant elements such as carbon or hydrogen. Surprisingly, linear regression performed better for iodine in the ATB dataset than the corresponding random forest model (see **Supplementary Material**). A possible explanation for that is the fact that iodine atoms are the most underrepresented elements in the ATB dataset, and the random forest models tend to overfit.

Generally, as **Table 1** shows, our predictions for the PRODRG dataset are more accurate than for ATB. There are several possible reasons for that. First, PRODRG is based on a simpler method for assigning partial charges (Altman, 1992). Second, we used molecules from the PubChem database for the PRODRG dataset. The three-dimensional structures of these molecules are all idealized and normalized by PubChem (Bolton et al., 2008). Compared to that, we used curated molecules for the ATB dataset, which mostly originate from the manually curated ChEMBL database (Gaulton et al., 2012; Stroet et al., 2018). Third, ATB performs geometric optimization and remaps the partial charges back to the original structures. Geometry-optimized charges cannot be learned by our model since we do not take geometrical temporary changes into account. Additionally, as shown in **Figure 3**, the partial charges for the ATB data have a higher variance, which makes prediction generally more difficult.

Although our approach is biased to inherit errors from the original tools, the predictions achieve a reliable approximation with low RMSE values. Inconsistent partial charges, which can appear in PRODRG (Lemkul et al., 2010), are unlikely because our models predict the charges along with defined models without determinations of building blocks. Error propagation cannot be avoided; however, by using larger datasets and extended feature sets, the prediction models tend to be more accurate. Our web tool is freely accessible at http://contradrg.heiderlab.de.

## Conclusion

All existing approaches of partial charges predictions for molecules aim at reconstructing the exact empirical-validated value. Thus, the computations are based on empirical determined data (Mortier et al., 1986; Besler et al., 1990) or on quantum mechanical theories (Manz and Sholl, 2010; Manz and Sholl, 2012; Manz and Limas, 2016). However, our approach tries to emulate the algorithm of the predictor without implementing any background knowledge about the underlying theories. Analysis of the input and output data from the web servers with subsequent machine learning approaches are sufficient to easily compute reliable approximations. Our web tool can be used to assign partial charge predictions automatically within seconds. This allows, for example, the correction of precalculated topology files. In the future, we intend to improve our models by using more training data, in particular for those atoms that are underrepresented, and to extend the feature set. Additionally, we intend to generate GROMOS-compatible topology files without geometrical optimization for molecular dynamics simulations.

## Data Availability Statement

The datasets generated for this study can be found in http://cdrg.mathematik.uni-marburg.de/data/raw-dataset.zip.

## Author Contributions

RM performed the data and machine learning analysis. RM drafted the manuscript. DH supervised the project, discussed the results, and revised the manuscript. All authors read and approved the final manuscript.

## Funding

This study was funded by the European Regional Development Fund, EFRE-Program, European Territorial Cooperation (ETZ) 2014-2020, Interreg V A, Project 41.

## Conflict of Interest

The authors declare that the research was conducted in the absence of any commercial or financial relationships that could be construed as a potential conflict of interest.
