# Supplementary material for: ContraDRG: Automatic Partial Charge Prediction by Machine Learning
Source: Front Genet. 2019 Oct 30;10:990. doi: 10.3389/fgene.2019.00990 (PMC6831742; doi:10.3389/fgene.2019.00990)
Supplement: Supplementary file 1 [file DataSheet_1.pdf]

Supplementary Material

1 SOFTWARE FOR PARTIAL CHARGE PREDICTION

In table S1 , we provide a comparison between existing tools for partial charge predictions.

Table S1. Comparison of different software tools for partial charge predictions in terms of high-quality quantum mechanical calculation and molecular dynamics (MD) force field compatible accuracy (Acc). ATB and PRODRG are proprietary software but provide a free access for academic purposes. We considered the ATB DFT Hessian QM calculation for this comparison. -, 0, and + indicate the relative performance in terms of accuracy and execution time, respectively. For the MD simulations, + and - indicate whether the software is suitable for molecular dynamics.

Software	License	Acc	Time	MD
Antechamber	Free	0	+	+
ATB	Proprietary (a)	+	-	+
PRODRG	Proprietary (a)	-	+	+
AutoDockTools	Free	-	+	-
OpenBabel	Free	-	+	-
VeraChem‘s Vcharge	Proprietary	0	+	+
Schroedinger Maestro	Proprietary	0	+	+
**ContraDRG**	**Free**	**+**	**+**	**+**

2 MACHINE LEARNING

Several machine learning algorithms were applied on both datasets (ATB and PRODRG) resulting in multiple models. Generally, models based on the most abundant elements perform better.

All applied parameters, hyperparameters, and the data set sizes are shown in table S2 and S3. The table S4 and S5 represent all machine learning performances on predicting partial charges.

Table S2. Machine learning parameters and hyperparameters for the PRODRG-derived models. Best settings for gradient boosting machine (**GBM**), linear regression (**LM**), k-nearest neighbors (**KNN**), random forest (**RF**), quantile random forest (**qRF**), and support vector machines (**SVM**) with various kernels, such as linear (**SVM L**) and radial (**SVM R**), are shown.

	element	C	H	N	O	P
	train set	70591	4748	7388	12875	247
	test set	23529	1581	2460	4290	80
GBM	n.trees	150	150	150	150	150
	interaction.depth	3	3	3	3	3
	shrinkage	0.1	0.1	0.1	0.1	0.1
	n.minobsinnode	10	10	10	10	10
LM	Intercept	True	True	True	True	True
KNN	kmax	9	5	5	7	5
	distance	2	2	2	2	2
	kernel	1	1	1	1	1
RF	mtry	35	68	35	35	35
qRF	mtry	68	68	68	68	68
SVM L	C	1	1	1	1	1
SVM R	sigma	0.0128	0.0133	0.0114	0.0132	0.0146
	C	1	0.25	1	1	1
	element	S	F	Cl	Br	I
	train set	826	564	1363	336	722
	test set	273	186	452	109	239
GBM	n.trees	150	150	150	150	150
	interaction.depth	3	3	3	3	3
	shrinkage	0.1	0.1	0.1	0.1	0.1
	n.minobsinnode	10	10	10	10	10
LM	Intercept	True	True	True	True	True
KNN	kmax	5	7	5	5	5
	distance	2	2	2	2	2
	kernel	1	1	1	1	1
RF	mtry	68	68	68	35	68
qRF	mtry	35	68	68	68	68
SVM L	C	1	1	1	1	1
SVM R	sigma	0.012	0.0281	0.0315	0.0467	0.047
	C	1	1	1	1	1

Table S3. Machine learning parameters and hyperparameters for the ATB-derived models. Best settings for gradient boosting machine (**GBM**), linear Regression (**LM**), k-nearest neighbors (**KNN**), random forest (**RF**), quantile random forest (**qRF**), and support vector machines (**SVM**) with various kernels, such as linear (**SVM L**) and radial (**SVM R**), are shown.

	element	C	H	N	O	P
	train set	65066	87659	9661	19205	758
	test set	21686	29217	3218	6401	251
GBM	n.trees	150	150	150	150	150
	interaction.depth	3	3	3	3	1
	shrinkage	0.1	0.1	0.1	0.1	0.1
	n.minobsinnode	10	10	10	10	10
LM	Intercept	True	True	True	True	True
KNN	kmax	9	9	9	9	5
	distance	2	2	2	2	2
	kernel	1	1	1	1	1
RF	mtry	35	35	35	35	68
qRF	mtry	66	56	68	68	68
SVM L	C	1	1	1	1	1
SVM R	sigma	0.0086	0.0113	0.0088	0.01	0.0158
	C	1	0.5	1	1	0.5
	element	S	F	Cl	Br	I
	train set	1579	1187	891	242	50
	test set	524	394	295	80	15
GBM	n.trees	150	150	150	150	150
	interaction.depth	3	3	3	3	3
	shrinkage	0.1	0.1	0.1	0.1	0.1
	n.minobsinnode	10	10	10	10	10
LM	Intercept	True	True	True	True	True
KNN	kmax	5	2	5	7	5
	distance	2	2	2	2	2
	kernel	1	1	1	1	1
RF	mtry	35	35	35	35	68
qRF	mtry	68	68	68	68	68
SVM L	C	1	1	1	1	1
SVM R	sigma	0.0096	0.0102	0.0144	0.0164	0.0175
	C	1	0.25	1	0.25	0.25

Table S4. Results of the PRODRG-derived models. Prediction performance values are shown as **R**MSE, **N**RMSE, and R^2. Results for gradient boosting machine (**GBM**), linear Regression (**LM**), k-nearest neighbors (**KNN**), random forest (**RF**), quantile random forest (**qRF**), and support vector machines (**SVM**) with various kernels, such as linear (**SVM L**) and radial (**SVM R**), are shown.

		C	H	N	O	P	S	F	Cl	Br	I
GBM	R	0.044	0.010	0.170	0.120	0.003	0.053	0.005	0.009	0.015	0.008
	N	5.761	5.548	7.085	7.494	0.287	2.431	3.474	7.220	11.620	4.840
	R^2	0.828	0.836	0.876	0.840	1.000	0.997	0.985	0.864	0.619	0.840
LM	R	0.051	0.014	0.242	0.150	0.004	0.106	0.007	0.019	0.017	0.010
	N	6.724	7.856	10.091	9.314	0.344	4.848	5.469	14.408	12.710	6.506
	R^2	0.762	0.665	0.738	0.747	1.000	0.988	0.966	0.451	0.558	0.709
KNN	R	0.010	0.006	0.079	0.064	0.002	0.103	0.008	0.004	0.016	0.007
	N	1.357	3.487	3.279	3.963	0.219	4.694	5.905	2.813	12.037	4.310
	R^2	0.990	0.934	0.972	0.955	1.000	0.989	0.958	0.979	0.587	0.875
RF	R	0.011	0.005	0.048	0.051	0.002	0.015	0.003	0.004	0.011	0.004
	N	1.443	2.878	1.986	3.184	0.152	0.678	2.436	2.724	8.625	2.575
	R^2	0.989	0.955	0.990	0.971	1.000	1.000	0.993	0.980	0.791	0.955
qRF	R	0.016	0.008	0.086	0.092	0.004	0.027	0.006	0.005	0.022	0.008
	N	2.057	4.147	3.568	5.746	0.347	1.224	4.742	3.982	16.708	5.132
	R^2	0.979	0.913	0.968	0.913	1.000	0.999	0.975	0.960	0.452	0.842
SVM L	R	0.054	0.026	0.249	0.153	0.073	0.120	0.007	0.020	0.016	0.010
	N	7.073	13.924	10.374	9.494	7.157	5.454	5.184	15.293	12.222	6.592
	R^2	0.738	0.010	0.730	0.739	0.965	0.985	0.968	0.415	0.589	0.706
SVM R	R	0.050	0.025	0.137	0.098	0.090	0.086	0.003	0.010	0.013	0.007
	N	6.566	13.831	5.698	6.116	8.835	3.921	2.382	7.938	9.976	4.464
	R^2	0.786	0.026	0.918	0.892	0.955	0.995	0.993	0.834	0.725	0.872

Table S5. Results of the ATB-derived models. Prediction performance values are shown as **R**MSE, **N**RMSE, and R^2. Results for gradient boosting machine (**GBM**), linear Regression (**LM**), k-nearest neighbors (**KNN**), random forest (**RF**), quantile random forest (**qRF**), and support vector machines (**SVM**) with various kernels, such as linear (**SVM L**) and radial (**SVM R**), are shown.

		C	H	N	O	P	S	F	Cl	Br	I
GBM	R	0.155	0.041	0.154	0.068	0.147	0.082	0.021	0.038	0.034	0.039
	N	5.387	5.151	7.357	6.058	7.247	3.756	5.137	6.828	9.114	13.724
	R^2	0.802	0.900	0.852	0.767	0.603	0.974	0.841	0.836	0.761	0.821
LM	R	0.151	0.043	0.161	0.070	0.094	0.081	0.022	0.044	0.034	0.030
	N	5.255	5.453	7.677	6.204	4.626	3.712	5.290	7.950	9.156	10.712
	R^2	0.811	0.888	0.837	0.753	0.838	0.974	0.826	0.777	0.756	0.922
KNN	R	0.095	0.022	0.148	0.055	0.120	0.098	0.020	0.039	0.032	0.044
	N	3.293	2.731	7.089	4.906	5.906	4.499	4.827	7.083	8.654	15.605
	R^2	0.926	0.972	0.862	0.847	0.728	0.962	0.858	0.829	0.796	0.855
RF	R	0.069	0.018	0.113	0.047	0.075	0.068	0.017	0.030	0.033	0.036
	N	2.398	2.313	5.391	4.200	3.712	3.095	4.179	5.490	8.796	12.840
	R^2	0.961	0.980	0.919	0.887	0.892	0.982	0.897	0.895	0.778	0.888
qRF	R	0.145	0.040	0.186	0.080	0.131	0.103	0.029	0.058	0.056	0.060
	N	5.037	4.989	8.869	7.090	6.461	4.722	7.035	10.589	14.971	21.298
	R^2	0.896	0.945	0.860	0.804	0.848	0.974	0.800	0.762	0.606	0.849
SVM L	R	0.152	0.046	0.163	0.071	0.097	0.087	0.037	0.054	0.049	0.062
	N	5.268	5.794	7.772	6.302	4.803	3.962	9.205	9.796	13.033	22.082
	R^2	0.810	0.879	0.834	0.746	0.823	0.971	0.520	0.705	0.531	0.624
SVM R	R	0.122	0.043	0.144	0.066	0.144	0.095	0.037	0.059	0.050	0.067
	N	4.228	5.423	6.888	5.830	7.108	4.338	9.004	10.676	13.202	23.767
	R^2	0.877	0.895	0.870	0.785	0.642	0.967	0.534	0.606	0.592	0.669

3 DIRECT COMPARISON

3.1 Partial charge comparison

Figure S1 shows a side-by-side comparison of the atomic partial charges for hydroquinone to illustrate the estimations of ContraDRG. Overall, ContraDRG performs as well as ATB 3.0 in this example. ATB 3.0 achieves an R^2 of 0.9899 compared to the quantum mechanical model, while ContraDRG achieves an R^2 of 0.9890.

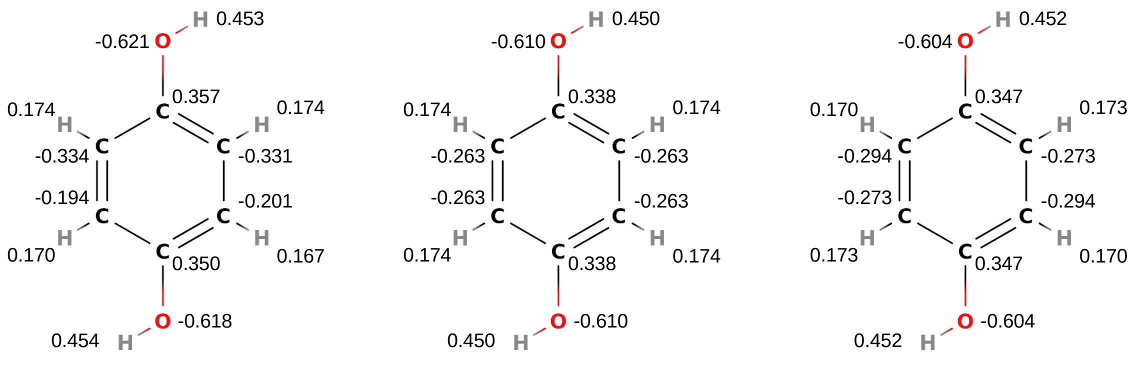

Figure S1. The partial charges of hydroquinone from quantum mechanical (QM) calculations according the B3LYP/6-31G* level of theory using the Kollman-Singh method [1; 2]. ATB 3.0 partial charges prediction according to the current ATB 3.0 database and the corresponding *ContraDRG* prediction.

3.2 Execution time

We performed a direct comparison between ATB and ContraDRG with respect to the execution time. Therefore, we choose random molecules from the existing ATB database [3] and uploaded the SMILEs into our ContraDRG web application.

Table S6. Comparison between the execution time (in seconds) of ten randomly chosen molecules for ATB 3.0 and ContraDRG, respectively.

molecule-ID	Execution time (s)		number of atoms
	ATB 3.0	ContraDRG	
3443	34,757	9.2	25
4050	30,922	7.3	21
10953	423,780	7.6	20
15175	87,835	13.6	39
19526	69,522	9.0	24
22917	32,833	13.0	34
30913	237,436	11.9	33
31995	25,950	7.5	21
37542	8,827	4.9	12
39702	9,552	6.3	17

4 MOLECULAR DYNAMICS

We performed replicated molecular dynamics simulations with GROMACS (v. 2016.3) with 50 randomly chosen molecules from the ATB database to calculate ΔG hydration free energy and compare it to experimentally determined values. Results are shown in figure S2. For that purpose, we used ATB topology

and molecule files. Further, we replaced the partial charges with those from ContraDRG. The average kJ/mol was used for comparison (see table S7).

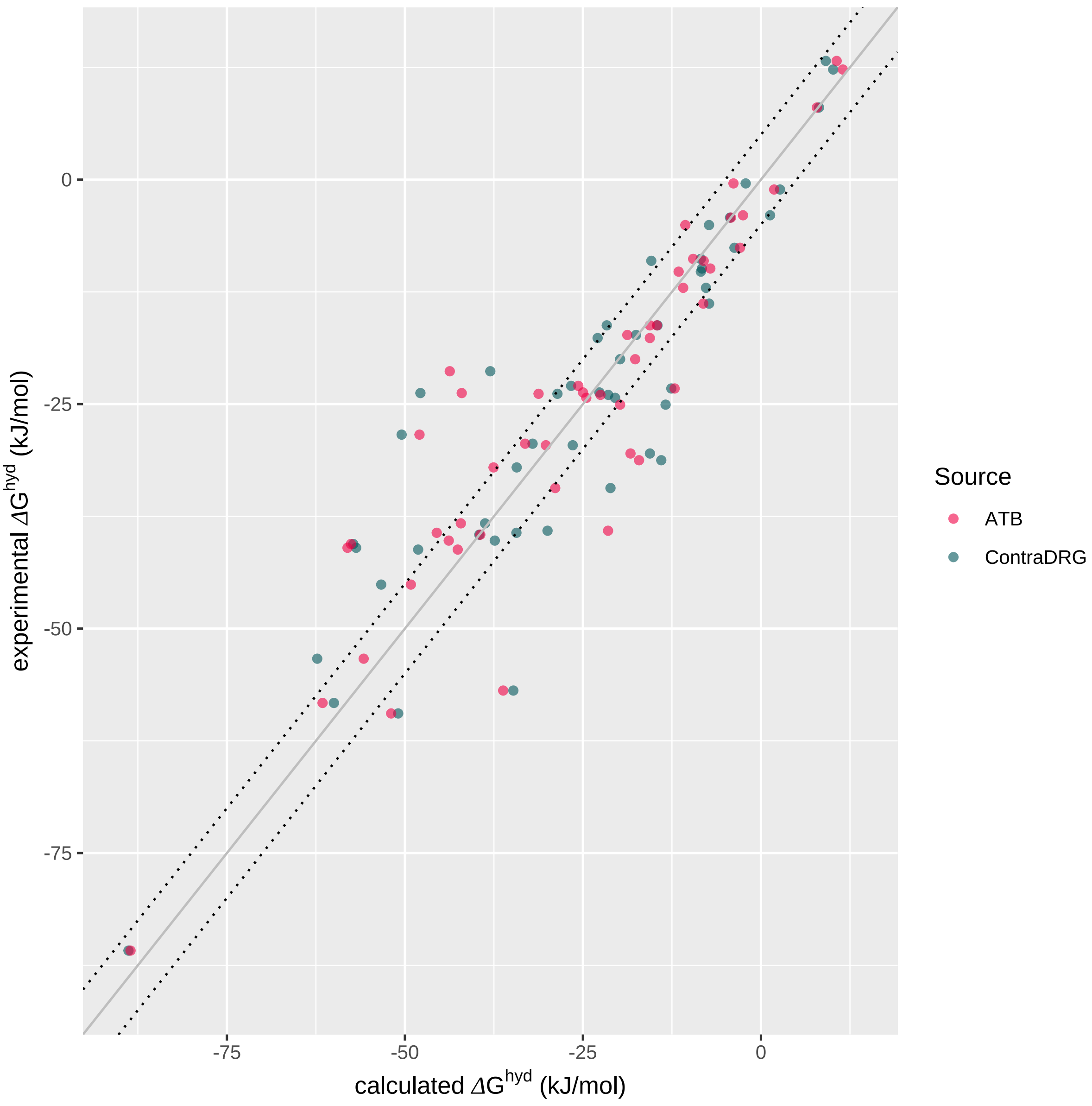

Figure S2. ΔG^{hyd} free energy values of 50 randomly chosen small molecules. Original partial charges from ATB versus predicted partial charges from ContraDRG (ATB-derived) models are shown. The dotted lines marks the ± 5.0 kJ/mol area.

There are no significiant differences between ATB and ContraDRG according to the Welch's two-sample t-test ($p = 0.53$). ATB and ContraDRG achieve $R^2 = 0.84$ and $R^2 = 0.82$, respectively, compared to the experimental results. ContraDRG obtains an $R^2 = 0.96$ for the ATB predictions.

Table S7. Molecules are taken from the ATB database (ID). Comparison of ΔG^{hyd} free energy results (kJ/mol) with partial charges derived from ATB and ContraDRG (cDRG). Experimental values (Exp.) ± error (error), as well as average predictions by ATB and ContraDRG (cDRG) ± standard deviations (SD) are shown. *: no errors are provided.

ID	Exp. ± error	ATB ± SD	cDRG ± SD
648	-30.50 ± 2.50	-18.32 ± 0.11	-15.59 ± 0.21
829	-16.23 ±2.51	-15.60 ± 0.14	-14.53 ± 0.23
870	-7.59*	-2.94 ± 0.12	-3.72 ± 0.10
881	-10.25 ± 0.84	-11.56 ± 0.16	-8.44 ± 0.09
892	-12.05 ± 2.51	-10.92 ± 0.16	-7.72 ± 0.16
962	-8.83 ± 2.51	-9.52 ± 0.19	-8.45 ± 0.19
1058	12.26 ± 2.51	11.50 ± 0.17	10.14 ± 0.15
1178	-4.23 ± 2.51	-4.21 ± 0.13	-4.34 ± 0.09
1189	-29.41 ± 2.51	-33.12 ± 0.12	-32.07 ± 0.16
1194	-1.10 ± 2.50	1.85 ± 0.14	2.68 ± 0.15
1918	-41.00 ± 8.00	-58.06 ± 0.28	-56.85 ± 0.17
1920	-39.54 ± 0.42	-39.41 ± 0.21	-39.55 ± 0.15
1939	-25.06 ± 0.42	-19.78 ± 0.14	-13.39 ± 0.09
1959	-39.33 ± 4.18	-45.53 ± 0.22	-34.34 ± 0.17
2589	13.22 ± 2.51	10.63 ± 0.23	9.11 ± 0.17
2809	-9.04 ± 0.42	-8.04 ± 0.14	-15.40 ± 0.15
3186	-0.42 ± 0.84	-3.86 ± 0.11	-2.14 ± 0.12
7966	-23.70 ±0.40	-24.99 ± 0.09	-22.69 ± 0.12
8019	-20.00 ± 1.05	-17.66 ± 0.17	-19.78 ± 0.22
8039	-39.10 ± 2.60	-21.47 ± 0.16	-29.97 ± 0.24
8542	-23.26 ± 0.84	-12.12 ± 0.10	-12.59 ± 0.17
18185	-53.35 ± 4.18	-55.79 ± 0.23	-62.31 ± 0.22
19047	-23.85 ± 0.84	-31.23 ± 0.27	-28.58 ± 0.38
19765	-56.90 ± 0.84	-36.20 ± 0.25	-34.77 ± 0.14
24214	-9.90 ± 0.00	-7.11 ± 0.12	-8.29 ± 0.16
24510	-24.30 ± 0.40	-24.53 ± 0.14	-20.48 ± 0.19
25060	-23.77 ± 4.18	-42.02 ± 0.33	-47.82 ± 0.40
27176	-59.45 ± 4.60	-51.93 ± 0.17	-50.94 ± 0.25
29254	-28.40 ± 0.40	-47.95 ± 0.19	-50.46 ± 0.35
30190	-13.81 ± 2.51	-8.10 ± 0.18	-7.28 ± 0.19
30220	-3.97 ± 2.51	-2.52 ± 0.15	1.28 ± 0.12
30307	-5.06 ± 2.51	-10.62 ± 0.18	-7.29 ± 0.12
30308	-29.58 ± 5.73	-30.20 ± 0.20	-26.43 ± 0.24
30352	-34.35 ± 1.21	-28.89 ± 0.18	-21.13 ± 0.16
30372	-23.97 ± 0.42	-22.54 ± 0.14	-21.45 ± 0.11
30397	-40.20 ± 1.30	-43.84 ± 0.20	-37.38 ± 0.18
30418	-17.64 ± 2.51	-15.60 ± 0.14	-22.94 ± 0.10
30428	8.03 ± 2.51	7.85 ± 0.13	8.15 ± 0.11
30441	-22.97 ± 2.51	-25.65 ± 0.15	-26.66 ± 0.17
30469	-16.23 ± 2.51	-14.61 ± 0.16	-21.65 ± 0.13
30471	-17.30 ± 5.70	-18.77 ± 0.15	-17.54 ± 0.12
30491	-32.05 ± 2.51	-37.55 ± 0.29	-34.30 ± 0.30
30547	-58.28 ± 4.18	-61.56 ± 0.36	-59.97 ± 0.28
30624	-21.34 ± 0.84	-43.70 ± 0.38	-38.01 ± 0.20
30652	-38.28 ± 4.18	-42.14 ± 0.20	-38.74 ± 0.28
32374	-85.86 ± 1.10	-88.53 ± 0.18	-88.82 ± 0.45
48262	-31.25 ± 2.51	-17.12 ± 0.08	-14.00 ± 0.10
103297	-41.20 ± 0.40	-42.58 ± 0.20	-48.14 ± 0.18
121093	-40.58 ± 4.18	-57.58 ± 0.16	-57.25 ± 0.15
228665	-45.10 ± 0.75	-49.16 ± 0.20	-53.33 ± 0.29

5 WEB APPLICATION

ContraDRG can be used in two different ways of operation: i) by using the website and ii) through a direct HTTP/HTTPS request, which is more suitable for automated file processing. ContraDRG processes only one molecule per submission except for multi-line SMILE submission through the website.

After processing the file, the user will obtain a unique result id, independently of the chosen submission method. Website users will additionally obtain one or up to ten molecule ids, depending on the number of submitted molecules. By using the result id, output files are directly accessible, see figure S3. If available, a molecule id should be specified for retrieving the output files and a format code, which is noted in table S8. Otherwise, ContraDRG will assume the first associated molecule of result id (submission) and the all atoms-PDB format. Generally, all results can be viewed on the results overview page.

Table S8. Supported output file formats and codes for the ContraDRG. All PDB files contain an additional column with the predicted partial charges.

Code	Format	Atom model	Note
pdb	PDB	All atoms	Including all hydrogens
pdbn	PDB	United atoms	Summarized and re-balanced partial charges
pdbqt	PDBQT	United atoms	Summarized and re-balanced partial charges, AutoDock 4 atom-types and torsions

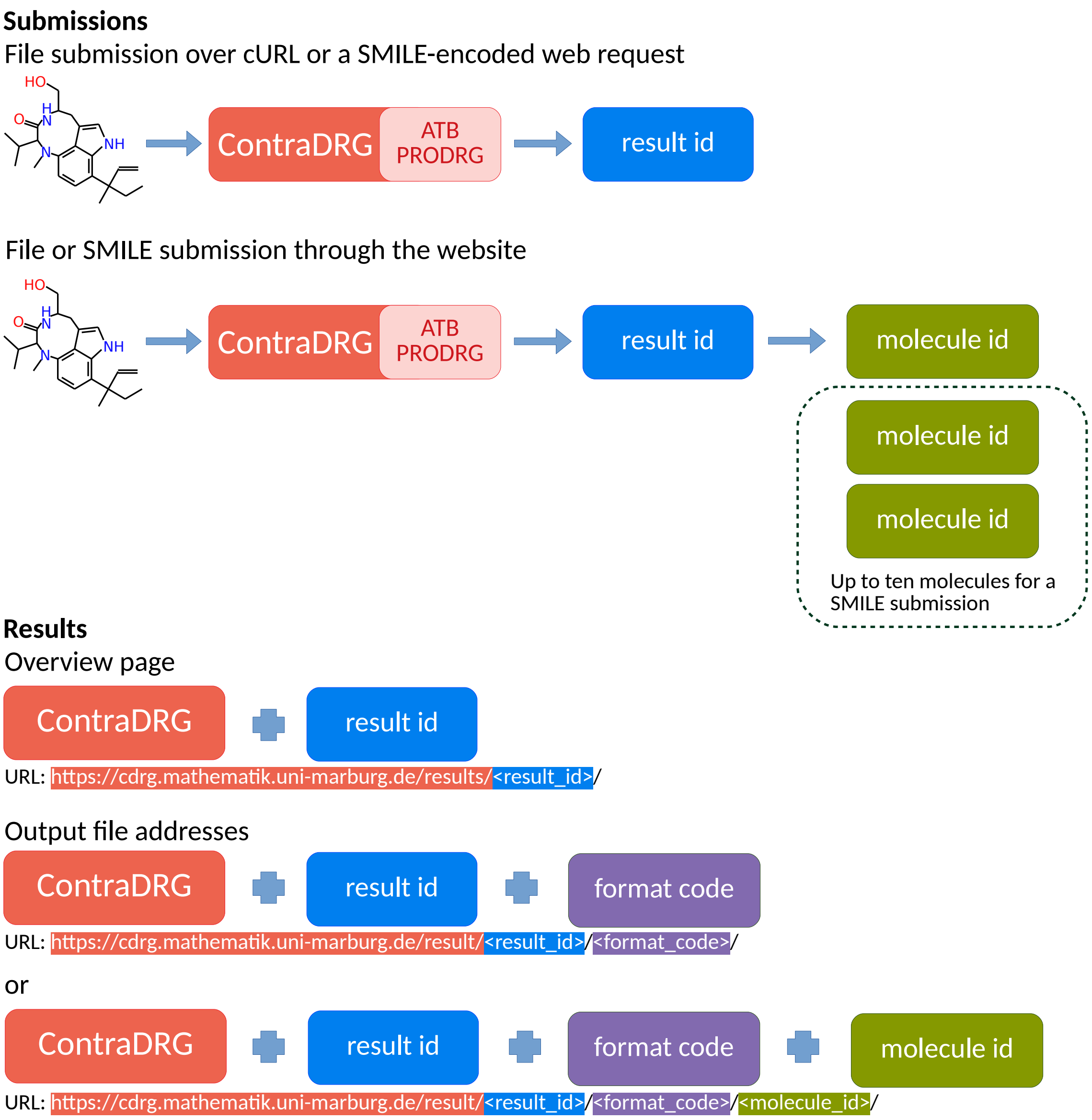

Figure S3. Submissions: Molecules can either be submitted as a molecule file (pdb/mol2/sdf/smi) over cURL, as a SMILE-encoded web request for automated processing, or through the website interface. After finishing the predictions, the request will be re-directed to the results page, including a result id. Submissions by using the website will lead to an overview page with a result id and a list of processed molecules, including individual molecule ids. **Results:** Processed molecule files can be accessed by the overview page or by a request including the result id, the desired output file format, or in case of multiple input submission with the additional molecule id. A list of supported file format codes is shown in table S8.

REFERENCES

[1]Singh UC, Kollman PA. An approach to computing electrostatic charges for molecules. *Journal of Computational Chemistry* **5** (1984) 129–145. doi:10.1002/jcc.540050204.

[2]Malde AK, Zuo L, Breeze M, Stroet M, Poger D, Nair PC, et al. An Automated force field Topology Builder (ATB) and repository: Version 1.0. *Journal of Chemical Theory and Computation* **7** (2011) 4026–4037. doi:10.1021/ct200196m.

[3]Stroet M, Caron B, Visscher KM, Geerke DP, Malde AK, Mark AE. Automated Topology Builder Version 3.0: Prediction of Solvation Free Enthalpies in Water and Hexane. *Journal of Chemical Theory and Computation* **14** (2018) 5834–5845. doi:10.1021/acs.jctc.8b00768.
